# Electrochemical Skin Conductance Alterations during Spinal Cord Stimulation: An Experimental Study

**DOI:** 10.3390/jcm10163565

**Published:** 2021-08-13

**Authors:** Lisa Goudman, Nieke Vets, Julie Jansen, Ann De Smedt, Maxime Billot, Philippe Rigoard, Ann Cordenier, Sebastiaan Engelborghs, Aldo Scafoglieri, Maarten Moens

**Affiliations:** 1 Department of Neurosurgery, Universitair Ziekenhuis Brussel, Laarbeeklaan 101, 1090 Brussels, Belgium; Nieke.Vets@uzbrussel.be (N.V.); Julie.Jansen@uzbrussel.be (J.J.); Maarten.Moens@uzbrussel.be (M.M.); 2 Stimulus Consortium (Research and Teaching Neuromodulation Uz Brussel), Universitair Ziekenhuis Brussel, Laarbeeklaan 101, 1090 Brussels, Belgium; Ann.DeSmedt@uzbrussel.be; 3 Center for Neurosciences (C4N), Vrije Universiteit Brussel, Laarbeeklaan 103, 1090 Brussels, Belgium; Ann.Cordenier@uzbrussel.be (A.C.); sebastiaan.engelborghs@uzbrussel.be (S.E.); 4 Pain in Motion International Research Group, Laarbeeklaan 103, 1090 Brussels, Belgium; 5 Department of Physical Medicine and Rehabilitation, Universitair Ziekenhuis Brussel, Laarbeeklaan 101, 1090 Brussels, Belgium; 6 Prismatics Lab (Predictive Research in Spine/Neuromodulation Management and Thoracic Innovation/Cardiac Surgery), Poitiers University Hospital, 86021 Poitiers, France; Maxime.BILLOT@chu-poitiers.fr (M.B.); Philippe.RIGOARD@chu-poitiers.fr (P.R.); 7 Department of Spine, Neuromodulation and Rehabilitation, Poitiers University Hospital, 86021 Poitiers, France; 8 Institut Pprime UPR 3346, CNRS, ISAE-ENSMA, University of Poitiers, 86360 Chasseneuil-du-Poitou, France; 9 Department of Neurology, Universitair Ziekenhuis Brussel, Laarbeeklaan 101, 1090 Brussels, Belgium; 10 Reference Center for Biological Markers of Dementia (BIODEM), Institute Born-Bunge, University of Antwerp, Universiteitsplein 1, 2610 Antwerp, Belgium; 11 Experimental Anatomy Research Department, Vrije Universiteit Brussel, Laarbeeklaan 103, 1090 Brussels, Belgium; Aldo.Scafoglieri@vub.be; 12 Department of Radiology, Universitair Ziekenhuis Brussel, Laarbeeklaan 101, 1090 Brussels, Belgium

**Keywords:** autonomic nervous system, electrodermal activity, neuromodulation, chronic pain

## Abstract

Despite the well-known clinical effects of spinal cord stimulation (SCS), the mechanisms of action have not yet been fully unraveled. The primary aim of this study was to measure whether electrochemical skin conductance, as a measure of peripheral sympathetic autonomic function, is altered by SCS. A second aim was to compare skin conductance levels of patients with failed back surgery syndrome (FBSS) with age- and sex-matched healthy controls. Twenty-three patients with FBSS treated with SCS participated in this study. Sudomotor function was measured with the Sudoscan^TM^ instrument on the hands and feet during SCS on and off states. Difference scores in skin conductance between patients and age- and sex-matched healthy controls were calculated. Normal sudomotor function at the painful lower limb was revealed for 61% of the patients when SCS was activated. Skin conductance levels were not altered between on and off states of SCS. Differences in scores between patients and healthy controls were significantly different from zero. This study showed that SCS does not influencing the sympathetic nervous system in patients with FBSS, as measured by skin conductance levels. Moreover, it suggested that there is no normalization of the functioning of the sympathetic nervous system, despite the effectiveness of SCS to reduce pain intensity.

## 1. Introduction

In many patients with failed back surgery syndrome (FBSS), the origin of the persisting element in the pain experience is still unknown, which makes targeted treatment difficult to deliver [1,2]. Spinal cord stimulation (SCS) is a well-known treatment option for therapy-refractory FBSS, with pain-relieving effects as well as positive effects on quality of life, exercise capacity, anxiety, and depression [3,4,5,6,7,8,9]. Nevertheless, the exact mechanisms of SCS are not yet fully understood [10]. Since the late 1960s, with the introduction of the pain gate control theory [11], several complementary hypotheses based on spinal, segmental, and supraspinal elements have been proposed [12,13]. The hypotheses that have gained a lot of attention are the supraspinal mechanisms with modulations of the descending nociceptive inhibitory pathways [14], the ascending medial pathway [15], and the ascending lateral pathway [16,17]. More recent research has pointed towards the involvement of glial cells [18,19], gene expression [20], and local tissue temperature increases [21]. Another hypothesis is the influence of the autonomic nervous system in chronic neuropathic pain and the possible target focus on this part of the nervous system by SCS [22].

In healthy individuals, a dynamic balance between the excitatory sympathetic part and inhibitory parasympathetic part of the autonomic nervous system is present [23,24]. In chronic pain patients, dysregulation of the autonomic nervous system has been revealed, with a lower parasympathetic contribution compared to healthy controls [25,26]. These conclusions were based on analyses of heart rate variability, whereby high frequency oscillations can be denoted as mediated through the vagus nerve [27]. Previously, it was suggested that low-frequency oscillations represent a combination of sympathetic and vagal activity [28]; however, low-frequency power can also denote a measure of modulation of the cardiac autonomic outflow by baroreflexes and not a measure of cardiac sympathetic tone [29,30]. As such, there is a need for a more direct evaluation of the sympathetic nervous system.

Sudorimetry, i.e., measuring the function of sweat gland innervation, specifically evaluates the peripheral sympathetic system [31]. In contrast to quantitative sudomotor axon reflex tests or skin biopsies, the Sudoscan^TM^ is a simple, non-invasive sudomotor test that allows measurement of the sweat gland function [32]. It is easy to perform, has a good reproducibility [33,34], can be rapidly conducted, and is very well tolerated (i.e., not uncomfortable), which are crucial aspects to implement this technique in clinical practice [35]. This method is based on the electrochemical reaction between sweat chlorides and stainless steel electrodes in contact with the palms and soles [32]. In patients with diabetes mellitus, it has been denoted as a promising, sensitive tool to detect diabetic neuropathy, with proven diagnostic performance and usefulness to monitor treatment efficacy [34,35,36]. Currently, the Sudoscan^TM^ is used in a broad range of populations, including patients with COVID-19 [37], multiple system atrophy [38], and Parkinson’s disease [39].

The goal of this study was to further unravel the mechanisms of action of SCS by evaluating whether SCS can influence the peripheral sympathetic nervous system; therefore, the first aim was to measure whether electrochemical skin conductance, as measured with the Sudoscan^TM^, is altered by SCS in patients suffering from FBSS. A second aim was to compare skin conductance of patients with FBSS who receive SCS to an age- and sex-adjusted normal population.

## 2. Materials and Methods

### 2.1. Participants

Patients with FBSS who received treatment with SCS at the department of Neurosurgery of Universitair Ziekenhuis Brussels were invited to participate in this study. According to routine clinical care at our center (and regulated by reimbursement rules), all patients had a standard clinical visit regarding their treatment progress every 6 months. Selection of patients was based on those FBSS patients who were scheduled for a 6 month SCS follow-up visit. Patients were allowed to take part in this study if they were at least 18 years old. Patients were not allowed to take part in the study if they had impaired skin integrity at the fingers and feet or if they had been previously diagnosed with major psychiatric problems.

The study protocol was approved by the central ethics committee of Universitair Ziekenhuis Brussels (B.U.N. 1432020000073) on 27 May 2020. The study was registered on clinicaltrials.gov (NCT04668482). All patients provided written informed consent before participation. The study was conducted according to the revised Declaration of Helsinki (1998).

### 2.2. Protocol

This was an experimental study, consisting of a single outpatient visit. All patients who took part in this study were instructed to switch off SCS 12 h before their study visit. During the study visit, skin conductance was first recorded when SCS was still switched off. Afterwards, patients were asked to provide a pain intensity score. After this measurement, patients had to switch their neurostimulator on, followed by a rest period of 30 min. A second evaluation of skin conductance and pain intensity reporting was conducted after this 30 min break. Due to the pain-relieving effects of SCS, patients could not be blinded to the study conditions.

All patients were asked to confirm that they switched off SCS 12 h before the study visit. This statement was controlled by evaluating whether SCS was effectively switched off (which was the case for all patients) when patients presented themselves for the study visit.

### 2.3. Self-Reported Outcome Measurements

To assess the current pain intensity, the visual analogue scale (VAS) was used for lower back pain and leg pain separately. For the VAS, a 10 cm line on paper was provided to all patients, representing a continuum between no pain and maximal pain. Pain intensity is expressed here in mm on a scale from 0 to 100. Patients completed this questionnaire twice; once when SCS was switched off and once when SCS was activated. The VAS pain score is a reliable and valid tool that is sensitive to change [40,41,42,43].

The Medication Quantification Scale III (MQS) was used to quantify and monitor different drug regimens used to treat a variety of pain conditions [44]. This tool uses a numerical representation of the negative impact each medication has in treating a patient’s pain [45].

### 2.4. Sudomotor Function

The Sudoscan^TM^ (Impeto Medical, Paris, France) evaluates sudomotor function through the measurement of electrochemical skin conductance (ESC) using reverse iontophoresis and chronoamperometry [46]. It reflects the sudomotor skin reactivity, since sweat glands on the hands and feet are innervated by postglanglionic unmyelinated sudomotor cholinergic sympathetic C-fibers [37]. As such, it provides an indication of the peripheral sympathetic autonomic function [47]. This is a non-invasive method based on an electrochemical reaction between sweat chloride and stainless steel electrodes after stimulation of the sweat glands by a low voltage direct current (<4 V) [37]. At low voltages (<10 V), the stratum corneum is electrically insulating, and only sweat gland ducts are conductive [47]. During the examination, patients were asked to stand with the palms of their hands and soles of their feet in contact with the electrodes for 2–3 min. Electrochemical skin conductance is expressed here in microsiemens (μS) and was derived from the ratio between measured current and the applied voltage [37]. According to previously defined thresholds for Caucasians, >60 µS represents normal sudomotor function, 40–60 µS a moderate sudomotor dysfunction, and <40 µS a sudomotor severe dysfunction for ESC measured at the hands, while cut-off values of >70 µS, 50–70 µS, and <50 µS are applied for ESC at the feet, respectively [31]. All measurements took place in the same room to limit the influence of environmental factors. Patients were allowed 10 min to adjust before the start of the measurements to ensure all patients were feeling comfortable in the measurement room.

### 2.5. Reference Data

Reference data for ESC were taken from the study by Vinik et al. (2016), in which data from 1350 healthy study participants were collected and analyzed [48]. The selected normal data were stratified by age category, sex, and measurement location (hands or feet), which allowed matching with our study population.

### 2.6. Sample Size Calculation

Sample size calculation was performed using G*Power 3.1.3 (Düsseldorf, Germany) based on the sympathetic skin response of healthy controls versus patients with chronic lower back pain and FBSS [49]. Mean amplitude (mv) values of 5.27 (SD 2.47) for patients and 6.65 (SD 0.84) for healthy controls were used in the current calculation. The minimal total sample size for a Wilcoxon test with matched pairs should reach 20 patients, based on two-tailed testing with alpha = 0.05, a desired power of 0.85, and a correlation of 0.8 between the two conditions.

### 2.7. Statistical Analysis

All analyses were performed in R Studio version 1.2.5019 (R version 3.6), except for the mixed models, which were constructed with SAS version 9.4. Here, *p*-values of 0.05 or less were considered statistically significant. No missing data were observed, meaning there was no need for imputation strategies. Normality was evaluated with the Shapiro–Wilk test and QQ-plots, while equality of variance was evaluated with Levene’s tests. Descriptive statistics are provided as means (±SD) or as medians (first and third quartile). A simple regression model was built with pain intensity scores as the outcome variables and the on and off states of SCS and pain location (low back or leg) as explanatory variables using an automatic step function.

ESC values between SCS on and off states were compared with Wilcoxon tests for hands and feet separately. Additionally, mixed models were constructed with ESC values as outcome variables and SCS condition, age, MQS score, and pain intensity scores as exploratory variables. Individual difference scores between patients and age- and sex-matched healthy persons were calculated by subtracting the score of the matched control from the patient score. A negative difference score indicated that the patient score was lower than the score of the matched control. Single-sample Wilcoxon tests were applied to evaluate whether the difference scores were significantly different from zero.

## 3. Results

### 3.1. Descriptive Statistics

In total, 23 patients were included in this study. All experiments were conducted on 12 December 2020. Twelve males and 11 females took part in this study. Patients had a mean age of 55.0 (SD: 9.2) years and a median BMI of 27 (Q1–Q3: 26.0–29.5) kg/m^2^. The median duration that patients were implanted with SCS was 3 (Q1–Q3: 2–7) years. The median score on the MQS was 11 (Q1–Q3: 6.2–16.1). All patients were Caucasians.

**Figure 1:**
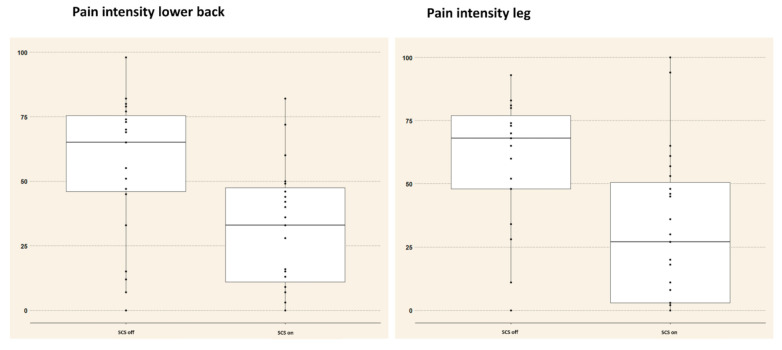
Boxplots showing individual observations of the pain intensity scores during SCS off and SCS on states for lower back (**left**) and leg pain (**right**). The first box presents the pain intensity scores during the SCS off state and the second box during the SCS on state. Abbreviations. OFF: SCS switched off; ON: SCS switched on; SCS: spinal cord stimulation.

Figure 1 presents boxplots with observed data points for pain intensity scores for lower back and leg pain separately (i.e., data as observed). A simple regression model for pain intensity scores revealed a significant effect of SCS condition (type III test: F = 20.32, *p* < 0.001) on pain intensity. The expected pain intensity score (i.e., based on the regression model) for a patient with FBSS when SCS was deactivated was 56.67 (95% CI from 48.89 to 64.46). When SCS was activated, the expected pain intensity score was 24.98 (95% CI from 13.97 to 35.99). Pain location (lower back or leg) was not withheld in the final model (type III test: F = 0.009, *p* = 0.92).

Additionally, 23 healthy controls were matched to the patients in our study population based on sex and age category.

### 3.2. Electrochemical Skin Conductance

The median ESC values did not significantly differ between SCS on and off states for measurements of the painful lower limb (V = 153.5, *p* = 0.19), non-painful lower limb (V = 114.5, *p* = 0.44), upper limb on the painful side (V = 147.5, *p* = 0.78), or upper limb on the non-painful side (V = 151, *p* = 0.70). The results of these measurements are presented in Table 1.

Mixed models for ESC on the lower limb on the painful side resulted in a significant effect of SCS condition (type III test F = 4.69, *p* = 0.04). The results of the model are presented in Table 2. Mixed models for ESC at the other locations did not result in significant type III tests.

During SCS, 14 (61%) patients showed normal sudomotor function, 7 (30%) patients presented with moderate sudomotor dysfunction, and 2 (9%) with severe sudomotor dysfunction with ESC recordings of the painful lower limb. Recordings for the hand (painful side) revealed 12 (52%) patients with normal sudomotor function, 10 (44%) with moderate sudomotor dysfunction, and 1 (4%) with severe sudomotor dysfunction during SCS.

### 3.3. Differences between Patients and Matched Controls

**Figure 2:**
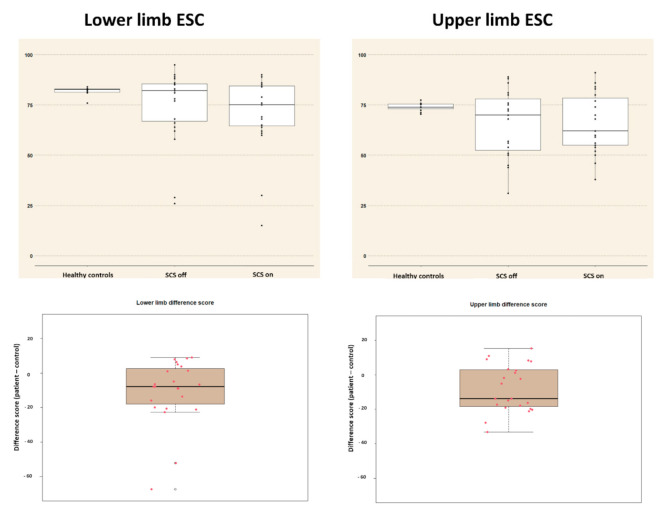
(**Upper row**) Boxplots of the electrochemical skin conductance values for healthy controls, for patients when SCS is switched off, and for patients when SCS is switched on for the painful side. (**Lower row**) Difference scores in electrochemical skin conductance for patients in the SCS on condition for healthy controls. For each figure, individual observations are plotted as well. OFF: SCS switched off; ON: SCS switched on; SCS: spinal cord stimulation.

The median ESC values for healthy controls were 82.70 (Q1–Q3: 81.35–82.90) for the lower limb and 73.80 (Q1–Q3: 73.05–75.50) for the upper limb, based on the data produced by Vinik et al. (2016) [48]. For patients, the median ESC values during SCS on the painful side were 75 (Q1–Q3: 64.50–84.50) for the lower limb and 62.00 (Q1–Q3: 55.00–78.50) for the upper limb. The median differences in ESC between patients during SCS and matched controls were −8.00 (Q1–Q3: −18.00–2.55) at the lower limb and −13.80 (Q1–Q3: −18.45–2.90) at the upper limb. The median difference scores were significantly different from zero for the lower limb (95% CI from −14.70 to −2.00, *p* = 0.009) and upper limb (95% CI from −15.55 to −2.40, *p* = 0.01). Figure 2 presents ESC values for healthy controls, patients during both SCS conditions, and difference scores.

## 4. Discussion

This study aimed to evaluate whether the autonomic nervous system, and more specifically the peripheral sympathetic system, was altered by SCS. By experimentally manipulating SCS conditions (SCS on versus off), the results indicated that skin conductance is not influenced by SCS. Additionally, skin conductance in patients with FBSS who were treated with SCS was compared to that of healthy controls. A statistically significant difference of −8.00 (Q1–Q3: −18.00–2.55) at the lower limb and −13.80 (Q1–Q3: −18.45–2.90) at the upper limb was found between both groups in favor of healthy controls. This indicates that in normal daily situations (SCS activated), the skin conductance of patients with FBSS was still different from the skin conductance of healthy controls, despite the treatment.

Previous evaluations of the functional impairment of non-myelinated postganglionic sudomotor sympathetic fibers in patients with chronic lower back pain and FBSS by measuring sympathetic skin response (SSR) revealed alterations in SSR (i.e., prolonged latency) in patients compared to healthy controls [49]. Prolonged SSR latencies were also found in another cohort of 29 patients with FBSS compared to healthy controls [50]. Moreover, in a cohort of 20 patients with FBSS, 18 out of 20 patients presented with abnormal neurophysiological findings compared to healthy controls, of which 35% demonstrated reduced SSR amplitudes in comparison to healthy control values [51]. Similarly, studies using the Sudoscan^TM^ to measure ESC indicated lower values in patients with fibromyalgia [52], Fabry disease [32], and psoriatic arthritis [47] compared to healthy controls. The study results indicated that despite providing SCS (a well-known effective treatment for this patient population [53]), ESC values for both lower and upper limbs were significantly different from the values for healthy controls. This suggests that despite treatment, there was no normalization of sudomotor function towards the level of healthy controls, despite the fact that sudorimetry is considered an efficient tool for treatment monitoring [31]. Indeed, the study by Calvet et al., (2013), in which 52 patients with type 1 diabetes and 115 patients with type 2 diabetes were followed for 12 months, indicated that an intensified insulin treatment resulted in improved ESC values [34]. Pain intensity reporting in our study clearly indicated that SCS was able to significantly reduce pain; nevertheless, this was not accompanied by an increase in ESC. It may be hypothesized that ESC values cannot be normalized after a short time period of 30 min (applied wash-in period for SCS in this study) and that longer treatments are needed, under the assumption that SCS improves ESC values.

Conversely, it is also plausible that SCS provided at the thoracic level does not influence the sympathetic system but only the parasympathetic system. Previous studies suggested that the presence of chronic pain induces changes in the balance of the autonomic nervous system [24,54]. Indirect evaluations relying on heart rate variability were suggestive of a reduction in the activity of the inhibitory parasympathetic system in patients with chronic pain [24]. This imbalance of the autonomic nervous system could be restored by applying SCS in patients with FBSS, as measured by an increase in high-frequency power (vagally mediated) during SCS compared to the situation without SCS [55,56]. Moreover, in a previous study in patients with FBSS, no influence of SCS was revealed based on skin conductance (measure of sympathetic activity), which was evaluated at the fingers (i.e., a remote location compared to the painful region) [56]. Additionally, in patients with chronic refractory angina, a reduction of the low-frequency/high-frequency ratio based on heart rate variability measurements was found when SCS was turned off [57,58], which is suggested to be a marker of sympathovagal balance, reflecting the relationship between sympathetic and parasympathetic components [59]. Instead of relying on indirect measurements of the functioning of the autonomic nervous system by heart rate variability recordings or on measurements at a remote region, the current study specifically evaluated peripheral sympathetic sudomotor activity in the painful region, without an influence of SCS. This leads to the hypothesis that if SCS is influencing the autonomic nervous system, it is predominantly inducing an effect on the parasympathetic system leading to a normalization of the disbalance between the sympathetic and parasympathetic system, without explicitly altering the functioning of the sympathetic system. A direct evaluation of the parasympathetic system could test this hypothesis and help us to further unravel the mechanisms of action of SCS. A review of experimental studies on the mechanisms behind SCS in vascular diseases proposed that SCS suppresses sympathetic activity to obtain SCS-induced vasodilation [60], a finding that could be accompanied with an active stimulation of the parasympathetic system by SCS.

Patients who were included in this study were implanted with SCS for 3 (Q1–Q3: 2–7) years. The initial effectiveness of SCS generally declines over time due to growing tolerance of the central nervous system in around 20–40% of patients [61,62]. If this is the case, SCS system explantation is often performed and a substantial proportion of these explants happens before 2.25 years of SCS treatment [63], the so-called “break even” point for SCS treatment when compared with conventional medical management [64]. Patel et al. (2019) reviewed the reasons for SCS device explantation in a sample of 129 patients who were treated with SCS [65]. The median time to explantation was 20 months (IQR 7.5–45.5 months), with the primary reason being a lack of efficacy (80.6%) [65]. Only patients who had not yet undergone a device explantation were eligible in this study. This indicates that the obtained study results (i.e., no normalization of skin conductance in patients with FBSS compared to healthy controls and no alteration in skin conductance when experimentally manipulating SCS status) are only valid for patients who are still receiving SCS. Similar (negative) results can be expected at shorter time periods, assuming that SCS is not inducing transient effects on the autonomic nervous system. Additionally, it might be expected that the lack of normalization of skin conductance to the level of healthy controls will also be present in patients who have undergone device explantation. Nevertheless, future studies are needed to confirm these hypotheses.

In this study, the Sudoscan^TM^ was used to evaluate sweat gland function by measuring sweat chloride concentrations using reverse iontophoresis and chronoamperometry [47], without the need for wetting of the skin. In general, wetting or occlusion of the skin by the recording electrolyte can significantly influence EDA [66] in the sense that a dry corneum conducts electricity poorly, whereby eventually a premium is placed on surface sweating, whereas conductivity increases with higher degrees of hydration [67] (i.e., different electrical conductivity [68]). Additionally, the role of skin blood flow could be evaluated in relation to skin conductance; especially for ischemic pain, SCS most likely affects skin blood flow [69]. On the other hand, in patients with neuropathic pain, no change in peripheral skin blood flow was revealed due to SCS [70]. Recently, in 12 healthy persons, transcutaneous electrical SCS (non-invasive) was applied at T11–T12 and L1–L2 with evaluations of the shin skin [71]. A significant increase in skin blood flow was revealed with a proposed crucial role of the mediator nitric oxide [71]. By experimentally manipulating the SCS status, emotional or psychological states could be altered, which might be associated with alterations in skin blood flow [72,73], potentially influencing skin conductance measurements in this study. Finally, exposing the skin, and more specifically the stratum corneum, to high environmental humidity can promote the hydration of the stratum corneum, whereby high humidity exposure increases skin conductance and skin susceptance in healthy participants [74]; therefore, all measurements took place in the same room on the same day to limit the influence of environmental factors on skin conductance.

Previous research studies have already explored the electrochemical behavior of stainless steels (of which the electrodes of the Sudoscan are composed) by mimicking the behavior of electrodes in contact with sweat through stainless steel 304 L and synthetic buffer carbonate solutions containing chloride, lactate, and urea [75]. The results indicated that stainless steel 304 L is a suitable material for the assessment of sudomotor dysfunction due to its high ability to detect deviations in chloride concentration [75]. The sensitivity of stainless steel 304 L to chloride was higher than for nickel, which was previously used in measurement devices, with the disadvantage of a potential allergic reaction in some patients [76].

This study also had some limitations. Due to the longer wash-out period compared to the wash-in period for SCS, patients were not blinded to the stimulation conditions. An objective measurement instrument that does not rely on self-reporting of patients, i.e., Sudoscan^TM^, was used to limit the influence of the lack of blinding. Another limitation of this study is that the ESC data of patients with FBSS were compared with the ESC data from historical healthy controls. Finally, in this study, an experimental manipulation of the SCS status was performed in all patients. Skin conductance responses can be directly altered related to manipulating the device status or by indirect effects, such as increased pain intensity levels or alterations in psychological factors when SCS is switched off. The potential mediating effect of pain intensity or claims of causality cannot be made with the current study design.

## 5. Conclusions

Sudomotor function, as an indicator of peripheral sympathetic autonomic function, is not altered when manipulating SCS conditions in patients with FBSS. Moreover, the electrochemical skin conductance in patients with FBSS who are receiving treatment with SCS is different from skin conductance in healthy controls, indicating a lack of normalization in this population. The current findings lead to the hypothesis that SCS only influences the parasympathetic system, without a direct alteration of the sympathetic system.

## Figures and Tables

**Table 1:** Electrochemical skin conductance values (expressed in µS) obtained during SCS on and off conditions for patients with FBSS. Data are expressed as medians with Q1 and Q3. OFF: SCS switched off; ON: SCS switched on; SCS: spinal cord stimulation.

Location	SCS Off	SCS On	Test Statistic Wilcoxon Test
Foot, painful side	82.00 (Q1–Q3: 67.0–85.5)	75.00 (Q1–Q3: 64.5–84.5)	V = 153.5, *p* = 0.19
Foot, non-painful side	75.00 (Q1–Q3: 65.5–85.0)	70.00 (Q1–Q3: 59.0–85.5)	V = 114.5, *p* = 0.44
Hand, painful side	70.00 (Q1–Q3: 52.5–78.0)	62.00 (Q1–Q3: 55.0–78.5)	V = 147.5, *p* = 0.78
Hand, non-painful side	71.00 (Q1–Q3: 51.0–80.5)	62.00 (Q1–Q3: 52.5–76.5)	V = 151.0, *p* = 0.70

**Table 2:** Model estimates for electrochemical skin conductance on the lower limb on the painful side. The reference level for sex is male and the reference level for SCS is the SCS off condition. CI: confidence interval; MQS: Medication Quantification Scale III; OFF: SCS switched off; ON: SCS switched on; SCS: spinal cord stimulation; SE: standard error; VAS: visual analogue scale.

Explanatory Factor	Level	Estimate	SE	95% CI	Type III Test
Intercept		112.46	21.49	67.49 to 157.43	
Sex	female	−8.54	6.13	−21.38 to 4.30	F = 1.94, *p* = 0.18
SCS	on	−5.66	2.61	−11.13 to −0.19	F = 4.69, *p* = 0.04
Age		−0.60	0.37	−1.38 to 0.18	F = 2.60, *p* = 0.12
MQS score		0.44	0.32	−0.22 to 1.10	F = 1.94, *p* = 0.18
VAS score low back		−0.05	0.05	−0.17 to 0.06	F = 0.90, *p* = 0.35
VAS score leg		−0.05	0.05	−0.16 to 0.06	F = 0.83, *p* = 0.37

## Data Availability

The data presented in this study are available on motivated request from the corresponding author.
